# CosFNet: A Lightweight Epileptic EEG Detection Model Based on Cosine Convolution and FNet

**DOI:** 10.3390/bioengineering13070754

**Published:** 2026-06-27

**Authors:** Jiajun Tian, Yazhou Zhao, Weidong Zhou, Guoyang Liu

**Affiliations:** 1 School of Integrated Circuits, Shandong University, Jinan 250199, China; 2 Shenzhen Research Institute of Shandong University, Shenzhen 518000, China; 3 Tandon School of Engineering, New York University, New York, NY 10012, USA; 4 Key Laboratory of Social Computing and Cognitive Intelligence (Dalian University of Technology), Ministry of Education, Dalian 116024, China

**Keywords:** seizure detection, cosine convolution, FNet, Fourier transform, electroencephalography (EEG), lightweight neural network

## Abstract

**Background/Objectives:** Epilepsy is a prevalent chronic neurological disorder, and electroencephalography (EEG) remains essential for its diagnosis and long-term monitoring. Although deep learning-based automatic seizure detection has advanced considerably, existing models typically require extensive parameters and computational resources, limiting their deployment on resource-constrained platforms. **Methods:** In this study, we propose CosFNet, a hybrid lightweight architecture integrating cosine convolution with an FNet encoder, a Fourier-transform-based token-mixing encoder. The cosine convolution frontend parameterizes convolutional kernels with the cosine function to efficiently capture local spatiotemporal features. The FNet backend replaces traditional self-attention with a parameter-free two-dimensional discrete Fourier transform, enabling global mixing across temporal tokens and hidden feature dimensions with fast Fourier transform-based efficiency. With these advances, the model contains only 19,458 learnable parameters. **Results:** On the publicly available CHB-MIT dataset, CosFNet achieves a mean segment-level sensitivity of 97.60%, a specificity of 97.12%, an event-level sensitivity of 98.59%, a false detection rate (FDR) of 0.82/h, and an area under the receiver operating characteristic curve (AUC) of 97.87%. On our collected SH-SDU dataset, it attains a mean sensitivity of 92.87%, specificity of 94.74%, an event-level sensitivity of 99.41%, and an AUC of 96.29%. **Conclusions:** CosFNet achieves competitive detection performance with significantly low complexity, offering a viable pathway toward clinical deployment in resource-limited environments.

## 1. Introduction

Epilepsy is one of the most prevalent chronic neurological disorders, characterized by recurrent, unpredictable seizures resulting from abnormal hypersynchronous neuronal discharges [1]. It affects approximately 50 million individuals worldwide, with population-level prevalence estimates ranging from 4 to 10 per 1000 persons [2,3]. Epilepsy ranks among the leading neurological causes of disability-adjusted life years globally [4]. The sudden onset of seizures not only severely compromises quality of life but may also precipitate serious consequences including falls, drowning, and sudden unexpected death in epilepsy. Electroencephalography (EEG) constitutes the cornerstone tool for epilepsy diagnosis and monitoring, recording the synchronized electrical activity of neuronal populations to directly capture the spatiotemporal distribution of epileptiform discharges [5]. However, in long-term EEG monitoring, continuous recordings spanning hours to days require labor-intensive visual inspection by neurological experts, a process that is time-consuming and subject to substantial inter-rater variability. Consequently, the development of accurate and efficient automated seizure detection systems is of considerable clinical significance.

Over several decades, automated seizure detection has progressed substantially. In 1982, Gotman [6] pioneered an automated detection system based on half-wave decomposition and morphological rules, extracting amplitude, duration, and slope features from rhythmic EEG activity to identify seizure events, thereby inaugurating the era of computer-assisted EEG analysis. Subsequently, researchers introduced a range of handcrafted signal features for EEG classification. Subasi [7] combined discrete wavelet transform coefficients with a mixture-of-experts model for seizure classification. Yuan et al. [8] proposed an EEG classification approach based on the extreme learning machine and wavelet transform. Polat and Güneş [9] developed a hybrid system combining decision tree classifiers with fast Fourier transform features for epileptiform EEG classification. Li et al. [10] employed empirical mode decomposition together with common spatial patterns for seizure onset detection. Other studies explored power spectral density, nonlinear measures such as approximate entropy, and fractal dimensions as discriminative EEG features. These handcrafted features were typically combined with classical machine learning classifiers, including support vector machines, random forests [11], and multi-layer perceptrons. While these feature-engineering approaches demonstrated the discriminability of EEG features, they inherently depend on domain-expert prior knowledge for feature design, exhibit sensitivity to preprocessing pipelines, and suffer from limited generalization across patients and datasets.

Deep learning, as an advanced end-to-end learning paradigm, has demonstrated superior performance attributed to its hierarchical architecture and powerful automatic feature extraction capabilities [12,13]. By utilizing deep neural networks, seizure detection models can automatically learn discriminative representations directly from raw or minimally preprocessed EEG signals, substantially reducing dependence on manual feature engineering. Acharya et al. [14] first applied a 13-layer deep convolutional neural network (CNN) to EEG seizure detection, demonstrating the feasibility of deep learning in this domain. Ullah et al. [15] developed an automated epilepsy detection system using a deep learning approach that achieved competitive performance on multiple EEG datasets. Since then, one-dimensional CNNs [16], multi-scale CNNs, hybrid architectures integrating time–frequency transforms [17,18], and CNN classifiers with multi-domain feature fusion [19] have been widely adopted for automatic seizure detection.

Beyond convolutional approaches, recurrent neural networks have also been extensively investigated for EEG seizure detection due to their inherent ability to model temporal dependencies. Long short-term memory (LSTM) networks [20] and their variants, including bidirectional LSTM, have been widely used in seizure detection by incorporating multiple temporal features. Hu et al. [21] proposed a deep Bi-LSTM network for scalp EEG classification, achieving effective seizure detection through bidirectional sequence modeling. Tawhid et al. [22] combined convolutional layers with LSTM to simultaneously extract spatial and temporal features from EEG. Nie et al. [23] integrated fast Fourier transform with a fully convolutional nested LSTM for epilepsy classification. Anita and Kowshalya [24] proposed a multi-scale atrous-based deep CNN combined with LSTM for automatic seizure detection. Despite their enhanced ability to capture long-range temporal dependencies, recurrent models often suffer from high computational cost due to sequential processing, limiting their applicability in real-time scenarios.

However, conventional CNNs and recurrent architectures typically require deep structures and wide channel configurations to attain sufficient representational capacity, resulting in parameter counts from hundreds of thousands to millions. Such large models are prone to overfitting under the limited training data available per patient and impractical for resource-constrained wearable devices. Recent EEG seizure-detection methods can be understood along three representative lines. First, traditional pipelines extract handcrafted temporal, spectral, time–frequency, or spatial features and then use conventional classifiers such as support vector machines [7,10,11]. Second, convolutional neural network (CNN)-, recurrent neural network (RNN)-, and temporal convolutional network (TCN)-based deep models learn local waveform morphology and temporal context directly from segmented EEG, reducing dependence on handcrafted features but often increasing model size [25,26]. Third, Transformer- and attention-based models introduce global dependency modeling for long temporal contexts, but their self-attention modules can be computationally expensive for long-term EEG monitoring [27,28]. This progression motivates compact models that combine local rhythmic feature extraction with efficient global interaction. To address the efficiency challenge, researchers have explored lightweight architectures, including depth-wise separable convolution [29], residual connections [30], and group convolution [31], to reduce computation while maintaining representational power. An alternative direction explores structured kernel parameterization using analytic functions. Ravanelli and Bengio [32] proposed SincNet, constraining the first convolutional layer to parameterized sinc functions to improve filter interpretability. Alekseev et al. [33] introduced GaborNet with learnable Gabor filters in deep CNNs. Notably, Liu et al. [34] proposed cosine convolution, parameterizing each convolutional kernel via a cosine function with only two learnable parameters (amplitude and frequency), demonstrating remarkable parameter efficiency for EEG seizure detection. Subsequent work extended this paradigm to group cosine convolution for channel-wise spatial feature extraction [35], multi-scale cosine convolution for capturing heterogeneous epileptiform patterns [31], efficient quantization-aware deployment [36], and lightweight sleep apnea detection using single-lead electrocardiogram (ECG) [37].

It should be noted that SincNet and the cosine convolution used in this study serve different roles. SincNet typically parameterizes the first convolutional layer as sinc-based band-pass filters. In contrast, CosConv can replace convolutional kernels in multiple layers rather than only the first. This enables parameter reduction throughout the convolutional frontend, which is particularly advantageous when building compact multi-layer architectures for EEG seizure detection.

Concurrently, the Transformer architecture [38], leveraging self-attention for global dependency modeling, has rapidly expanded from natural language processing (NLP) to computer vision, exemplified by the Vision Transformer (ViT) [39], and time-series analysis. In EEG seizure detection, Sun et al. [27] applied a Transformer-based architecture for continuous seizure detection from long-term intracranial EEG. Zhang et al. [40] combined a bidirectional gated recurrent unit (GRU) with a Transformer encoder for knowledge-distillation-based detection. Wang et al. [28] proposed a CNN-ViT dual-branch network extracting complementary features from EEG spectrograms. Dutta et al. [41] employed a multi-head self-attention model for epilepsy identification from EEG. However, self-attention complexity of $O(L^{2}D)$ scales quadratically with input length, which is prohibitive for real-time processing of long-term EEG on resource-constrained devices [35]. To address this, Lee-Thorp et al. [42] proposed FNet, entirely replacing self-attention with a discrete Fourier transform (DFT) to achieve global token-feature mixing without learnable parameters in the mixing operation. FNet attains 92–97% of Bidirectional Encoder Representations from Transformers (BERT) accuracy on the General Language Understanding Evaluation (GLUE) benchmark while accelerating training by ∼80%. This demonstrates that deterministic Fourier transforms can effectively substitute self-attention for sequence modeling. Nevertheless, FNet has been investigated primarily in NLP, and its applicability to EEG biosignal analysis remains unexplored.

Motivated by these observations, this paper proposes CosFNet, a lightweight hybrid architecture for EEG-based seizure detection. The model employs cosine convolution as its frontend to efficiently capture local spatiotemporal EEG features, while incorporating an FNet encoder that performs parameter-free Fourier mixing across temporal positions and hidden features to compensate for the limited receptive field of shallow convolutions, with a lightweight feed-forward network (FFN) providing learnable nonlinear transformation. The CosFNet model contains only 19,458 learnable parameters and achieves detection performance comparable to existing methods on both the CHB-MIT dataset and the clinical SH-SDU dataset. CosFNet follows this compact local-to-global modeling strategy: the CosConv frontend acts as an oscillatory filter bank for local seizure-related EEG rhythms, whereas the FNet encoder provides parameter-free global mixing across the downsampled temporal positions and feature channels within each 4 s segment. Segment-level probabilities are subsequently converted into event-level detections by the postprocessing procedure described in Section 3.4. The main contributions of this study are summarized as follows:

1. We introduce the cosine convolution frontend to effectively capture local spatiotemporal features from epileptic EEG signals.
2. We integrate the parameter-free FNet with cosine convolution frontend to construct a compact end-to-end CosFNet model for EEG-based seizure detection.
3. We comprehensively evaluate the CosFNet on a publicly available CHB-MIT dataset and a clinically collected SH-SDU dataset. Compared with existing state-of-the-art methods, the proposed CosFNet achieves comparable performance with significantly fewer parameters.

The remainder of this paper is organized as follows. Section 2 details the proposed methodology. Section 3 describes the experimental datasets and preprocessing. Section 4 reports results. Section 5 presents ablation studies, feature visualization, and a comparison with existing methods. Section 6 concludes the paper.

## 2. Proposed Methods

**Figure 1:**
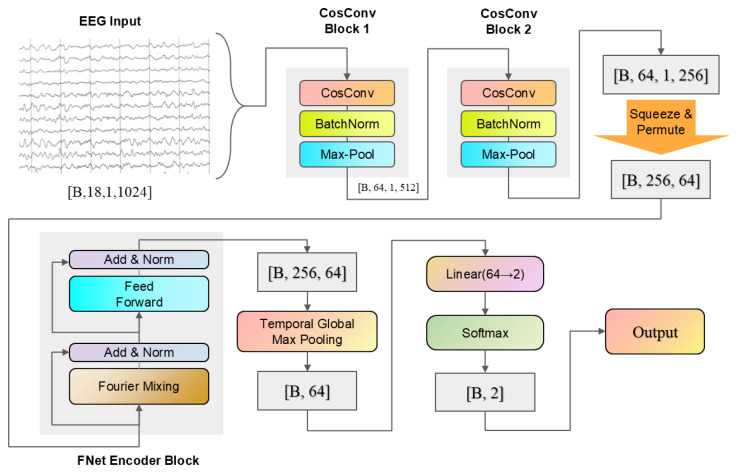
Overall architecture of CosFNet. The arrows indicate the direction of data flow; the orange arrow denotes the squeeze-and-permute operation that transforms the feature tensor from [B,64,1,256] to [B,256,64]. The model comprises two CosConv blocks for local feature extraction, an FNet encoder for global token-feature mixing, and a max-pooling classification head.

This section describes the proposed CosFNet framework for EEG seizure detection. As illustrated in Figure 1, the network first uses a shallow cosine convolutional frontend to extract local temporal patterns from multi-channel EEG segments, then applies an FNet encoder to mix information globally across downsampled temporal tokens and hidden feature dimensions, and finally produces a seizure probability through temporal max pooling and a linear classifier. In contrast to a deep CNN or an attention-based Transformer, the proposed design emphasizes a compact parameterization of local filters and a parameter-free global mixing operation, which aims to develop a lightweight model for resource-constrained monitoring scenarios.

### 2.1. Cosine Convolution Frontend

In a conventional one-dimensional convolutional layer, a kernel tensor $W\in R^{C_{out}\times C_{in}\times K}$ contains $C_{out}\times C_{in}\times K$ independently learnable weights. Such free-form parameterization increases rapidly with the kernel length, channel width, and network depth. Cosine convolution [34] reduces this redundancy by representing each temporal filter with a cosine function controlled by two learnable parameters, namely an amplitude *A* and an angular frequency ω. For a kernel of length *K*, a zero-centered coordinate vector is first defined as

(1) $$x=\left[-\frac{K-1}{2},\;-\frac{K-1}{2}+1,\;\ldots ,\;0,\;\ldots ,\;\frac{K-1}{2}\right]\in R^{K}.$$

The kernel connecting the *j*-th input channel to the *i*-th output channel is then synthesized as

(2) $$W_{ij}\left[m\right]=A_{ij}\cdot \cos\;\left(\omega_{ij}\cdot x\left[m\right]\right),\;m=0,1,\ldots ,K-1.$$

Here, $A_{ij}$ controls the filter gain, whereas $\omega_{ij}$ determines the oscillatory pattern and hence the frequency selectivity of the filter. The learnable parameter count of a cosine convolution layer is therefore

(3) $$P_{\mathrm{Cos}\mathrm{Conv}}=2\times C_{out}\times C_{in},$$

which corresponds to a ratio of 2/K relative to standard convolution. With the kernel length used in the final model (K=9), the convolutional kernel parameters are reduced to approximately 22.2% of those in a standard convolutional layer.

Equation (3) counts parameters at the level of individual input–output channel pairs and then sums over all pairs. Specifically, each pair (i,j) connecting the *j*-th input channel to the *i*-th output channel has two independently learnable parameters, $A_{ij}$ and $\omega_{ij}$. Therefore, the total number of learnable parameters is $2\times C_{out}\times C_{in}$. The operation is standard convolution rather than channel-wise convolution: each output channel owns $C_{in}$ cosine-parameterized filters, convolves them with the corresponding input channels, and sums the results to obtain the output waveform.

Given an input feature map $X\in R^{B\times C_{in}\times 1\times T}$, the cosine convolution layer dynamically synthesizes the kernel tensor using Equation (2) and applies convolution with same padding and unit stride:

(4) $$Z_{i}\left[t\right]=\sum_{j=1}^{C_{in}}\sum_{m=0}^{K-1}W_{ij}\left[m\right]\cdot {\tilde{X}}_{j}\left[t+m\right].$$

Substituting the cosine kernel yields

(5) $$Z_{i}\left[t\right]=\sum_{j=1}^{C_{in}}\sum_{m=0}^{K-1}A_{ij}\cos\;\left(\omega_{ij}\cdot x\left[m\right]\right)\cdot {\tilde{X}}_{j}\left[t+m\right],$$

where $\tilde{X}$ denotes the padded input sequence. The output feature map preserves the temporal length and has dimension $R^{B\times C_{out}\times 1\times T}$.

The two parameters of each cosine filter are optimized by standard backpropagation. For the loss function *E*, the derivative with respect to the amplitude is

(6) $$\frac{\partial Z_{i}\left[t\right]}{\partial A_{ij}}=\sum_{m=0}^{K-1}\cos\;\left(\omega_{ij}\cdot x\left[m\right]\right)\cdot {\tilde{X}}_{j}\left[t+m\right],$$

and, after accumulating over all temporal positions with $\delta_{i}\left[t\right]=\partial E/\partial Z_{i}\left[t\right]$,

(7) $$\frac{\partial E}{\partial A_{ij}}=\sum_{t=0}^{T-1}\frac{\partial E}{\partial Z_{i}\left[t\right]}\cdot \frac{\partial Z_{i}\left[t\right]}{\partial A_{ij}}=\sum_{t=0}^{T-1}\delta_{i}\left[t\right]\cdot \sum_{m=0}^{K-1}\cos\;\left(\omega_{ij}\cdot x\left[m\right]\right)\cdot {\tilde{X}}_{j}\left[t+m\right].$$

For the angular frequency parameter, the derivative of the synthesized kernel is

(8) $$\frac{\partial W_{ij}\left[m\right]}{\partial \omega_{ij}}=-A_{ij}\cdot \sin\;\left(\omega_{ij}\cdot x\left[m\right]\right)\cdot x\left[m\right],$$

which leads to

(9) $$\frac{\partial E}{\partial \omega_{ij}}=\sum_{t=0}^{T-1}\delta_{i}\left[t\right]\cdot \sum_{m=0}^{K-1}\frac{\partial W_{ij}\left[m\right]}{\partial \omega_{ij}}\cdot {\tilde{X}}_{j}\left[t+m\right]=-\sum_{t=0}^{T-1}\delta_{i}\left[t\right]\cdot \sum_{m=0}^{K-1}A_{ij}\sin\;\left(\omega_{ij}\cdot x\left[m\right]\right)\cdot x\left[m\right]\cdot {\tilde{X}}_{j}\left[t+m\right].$$

Because the frequency gradient contains the additional coordinate factor x[m], the amplitude and frequency parameters may have different gradient scales. Adam [43] is therefore used for optimization, and weight decay is not applied to ω to avoid driving the frequency parameters toward zero, which would degenerate the kernels into constant functions.

Each CosConv block consists of a cosine convolutional layer, batch normalization (BN), and temporal max pooling:

(10) $$\mathrm{Cos}\mathrm{ConvBlock}\left(X\right)={\mathrm{MaxPool}}_{1\times 2}\;\left(\mathrm{BN}\;\left(\mathrm{Cos}\mathrm{Conv}(X)\right)\right).$$

As shown in Figure 1, batch normalization stabilizes the channel-wise feature distribution,

(11) $${\hat{z}}_{i}\left[t\right]=\frac{z_{i}\left[t\right]-\mu_{i}}{\sqrt{\sigma_{i}^{2}+\epsilon }}\cdot \gamma_{i}+\beta_{i},$$

where $\gamma_{i}$ and $\beta_{i}$ are learnable scale and shift parameters. A 1×2 max-pooling operation with stride 1×2 halves the temporal length after each block. No additional explicit activation is inserted within the CosConv block. Nonlinear behavior is introduced by the max-pooling operation and by the Gaussian error linear unit (GELU) activation in the subsequent FNet feed-forward network.

### 2.2. FNet Encoder

Although self-attention is effective for modeling long-range dependencies, its $O(L^{2}D)$ complexity becomes costly for long temporal sequences. FNet [42] replaces the attention sublayer with a Fourier mixing operation, providing global interaction across temporal tokens and hidden feature dimensions through a deterministic transformation rather than learned attention weights. Given an input sequence $H\in R^{B\times L\times D}$, where *L* is the number of temporal tokens and *D* is the feature dimension, the Fourier mixing sublayer applies a two-dimensional DFT to each L×D sample matrix and retains the real part:

(12) FourierMixing(H)=ReF2D(H).

The 2D-DFT is defined as

(13) $$\hat{H}\left[u,v\right]=\sum_{l=0}^{L-1}\sum_{d=0}^{D-1}H\left[l,d\right]\cdot e^{-j2\pi (\frac{ul}{L}+\frac{vd}{D})}.$$

Each transformed position aggregates information from all temporal-token and hidden-feature positions, thereby allowing global token-feature mixing without introducing learnable parameters in the mixing operation. With fast Fourier transform (FFT) implementation, the computational complexity is O(LD(logL+logD)), substantially lower than the quadratic temporal scaling of self-attention.

The parameter-free Fourier mixing output is followed by an FFN, which provides learnable nonlinear transformation:

(14) $$\mathrm{FFN}\left(H^{\prime }\right)=\mathrm{GELU}\;\left(H^{\prime }W_{1}+b_{1}\right)W_{2}+b_{2},$$

where $W_{1}\in R^{D\times d_{ff}}$, $W_{2}\in R^{d_{ff}\times D}$, and $d_{ff}$ denotes the hidden dimension. GELU [44] is defined as

(15) GELU(x)=x·Φ(x),

where Φ(x) is the standard Gaussian cumulative distribution function. The complete FNet encoder block uses residual connections and layer normalization [45] around both the mixing and FFN sublayers:

(16) $$H^{\prime }=\mathrm{LayerNorm}\;\left(H+Y_{\mathrm{mix}}\right),$$

(17) $$H_{\mathrm{out}}=\mathrm{LayerNorm}\;\left(H^{\prime }+\mathrm{FFN}\left(H^{\prime }\right)\right).$$

For feature dimension *D* and FFN hidden dimension $d_{ff}$, the learnable parameter count of one FNet block, including two LayerNorm layers, is

(18) $$P_{\mathrm{block}}=2D\cdot d_{ff}+d_{ff}+D+4D=2D\cdot d_{ff}+d_{ff}+5D.$$

Table 1 summarizes the complexity difference between FNet and standard self-attention. By removing learned attention projections and the quadratic attention matrix, FNet is particularly suitable for compact EEG models that require efficient global token-feature mixing at low parameter cost, while its compact FFN preserves learnable nonlinear expressiveness. During inference, all model parameters remain fixed; the Fourier mixing operation is deterministic and does not depend on input-dependent attention weights.

### 2.3. CosFNet Architecture

CosFNet integrates the cosine convolution frontend and the FNet encoder into a single end-to-end seizure detection network. The input to the model is a 4 s multi-channel EEG segment $X_{0}\in R^{B\times 18\times 1\times 1024}$. The cosine convolution frontend contains *N* cascaded CosConv blocks, each of which extracts local temporal features and halves the temporal resolution:

(19) $$X_{n}={\mathrm{Cos}\mathrm{ConvBlock}}_{n}\left(X_{n-1}\right),\;n=1,\ldots ,N.$$

In the final configuration, N=2 and each cosine convolution layer has 64 output channels. The resulting feature map $H^{\left(N\right)}\in R^{B\times C_{N}\times 1\times T^{\prime }}$ is squeezed along the singleton spatial dimension and then permuted to yield the sequence representation $H_{\mathrm{seq}}\in R^{B\times T^{\prime }\times C_{N}}$, where the downsampled temporal positions serve as tokens and the convolutional channels serve as token features. The sequence is processed by *M* FNet encoder blocks,

(20) $$H_{\mathrm{out}}={\mathrm{FNetEncoder}}_{M}\;\left(H_{\mathrm{seq}}\right)\in R^{B\times T^{\prime }\times C_{N}},$$

where M=1 in the final model. The output sequence is summarized by temporal global max pooling,

(21) $$h=\max_{t=1}^{T^{\prime }}H_{\mathrm{out}}\left[:,t,:\right]\in R^{B\times C_{N}},$$

which retains the strongest response in each feature channel and is well matched to seizure-related EEG patterns that often occur as localized transient changes. A linear layer then maps h to two class logits,

(22) $$\ell =h\cdot W_{cls}+b_{cls}\in R^{B\times 2},$$

and the seizure probability is obtained by

(23) P(seizure∣X)=Softmax(ℓ)[:,1].

Table 2 reports the layer-wise configuration and parameter count. The final model contains two CosConv blocks with 64 channels, one FNet encoder block with $d_{ff}=64$, temporal max pooling, and a linear classifier. The total number of learnable parameters is 19,458, of which the cosine convolution frontend including batch normalization accounts for 55.3%, the FNet encoder including layer normalization accounts for 44.1%, and the classifier accounts for only 0.7%. The DFT mixing operation itself contributes no learnable parameters.

## 3. EEG Datasets and Experimental Setup

### 3.1. CHB-MIT Dataset

The first dataset employed in this study is the publicly available CHB-MIT scalp EEG dataset [5], collected at Boston Children’s Hospital and widely used in epilepsy seizure detection research. This dataset comprises multi-channel EEG recordings from 24 epilepsy patients (18 female, 5 male, 1 unknown, ages 1.5–22 years) acquired at a sampling rate of 256 Hz with 16-bit resolution. Electrodes were placed according to the international 10–20 system using a bipolar montage. The number of recorded channels varies from 18 to 26 across patients. To ensure input consistency, 18 common bipolar channels are selected: FP1-F7, F7-T7, T7-P7, P7-O1, FP1-F3, F3-C3, C3-P3, P3-O1, FP2-F4, F4-C4, C4-P4, P4-O2, FP2-F8, F8-T8, T8-P8, P8-O2, FZ-CZ, CZ-PZ.

The dataset contains approximately 979.93 h of EEG data with 184 expert-annotated seizure events across 24 patients. Patient 16 is excluded from this study due to extremely short seizure durations (mean 8.40 s), which provide insufficient training samples even after augmentation. Thus, 23 patients are used in all experiments. For each patient, only a small number of seizure events are included in the training set (typically $N_{t}=1$, increased to 3–4 for patients with very short individual seizures), while all remaining data constitute the test set. Table 3 summarizes patient-level details.

### 3.2. SH-SDU Dataset

The second dataset is a clinical scalp EEG dataset (SH-SDU), collected at the Second Hospital of Shandong University using the Natus NicoletOne EEG system (Nicolet v32 amplifier, Natus Medical Inc., CA, USA). This study was approved by the Ethics Committee of the Second Hospital of Shandong University (Approval No.: KYLL-2021CKJIP-0252). Data were recorded at 500 Hz in EDF format using a standard 10–20 system with 18 unipolar channels (Fp1, Fp2, F3, F4, C3, C4, P3, P4, O1, O2, F7, F8, T3, T4, T5, T6, A1, A2), with the reference electrode placed between Fz and Cz.

Compared with CHB-MIT, SH-SDU differs in both demographic composition and seizure density. It includes adult patients aged 28–79 years and contains a relatively larger number of seizures per patient, thereby providing complementary clinical recordings for evaluating the robustness of the proposed model. Eight patients (3 female, 5 male) with approximately 160.23 h of continuous EEG recordings and 170 expert-annotated seizure events are included. The same training procedure is applied. Table 4 summarizes patient details.

### 3.3. Preprocessing

A unified preprocessing procedure was applied to both datasets to obtain consistent model inputs. The CHB-MIT recordings were originally sampled at 256 Hz and were therefore used without resampling, whereas the SH-SDU recordings were downsampled from 500 Hz to 256 Hz using an anti-aliasing low-pass filter. After sampling-rate unification, a five-level discrete wavelet transform (DWT) with the Daubechies-4 (Db4) wavelet was applied to each EEG channel. Previous studies have demonstrated that the Db4 wavelet closely matches the morphological characteristics of epileptiform EEG discharges, and the DWT with Db4 has been widely adopted for preprocessing epileptic EEG signals [46,47]. The decomposition produced D1 (64–128 Hz), D2 (32–64 Hz), D3 (16–32 Hz), D4 (8–16 Hz), D5 (4–8 Hz), and A5 (0–4 Hz) components. The reconstruction of the D3, D4, and D5 detail components yielded a frequency band of interest (4–32 Hz) for subsequent analysis.

The filtered continuous EEG was segmented into non-overlapping 4 s segments (with the term “segment” being used instead of “epoch” to avoid confusion with training epochs in model optimization), corresponding to 1024 samples at 256 Hz. These segments served as the basic input units for testing. During training, ictal segments were generated with 80% overlap, equivalent to a stride of approximately 204 samples, to alleviate the scarcity of seizure samples. Non-ictal segments were extracted without overlap, and their total duration was set to approximately five times the original training-seizure duration. Because training ictal segments were augmented with about five-fold overlap, this setting produced a comparable number of non-ictal segments and augmented ictal segments for balanced model training. To ensure a consistent input dimensionality, CHB-MIT recordings were unified to 18 common bipolar channels using an electrode mask, whereas SH-SDU recordings were directly represented by their native 18 unipolar channels. The final input tensor was therefore arranged as (N,18,1,1024) in the NCHW format.

Table 5 compares key preprocessing parameters across the two datasets.

### 3.4. Training Settings

The training and testing seizure events are separated during preprocessing. Most patients use only one seizure event for training, whereas patients with short or frequent seizures use additional events to ensure sufficient ictal training samples. All remaining seizure events and interictal EEG data are reserved for testing. For CHB-MIT, 32 seizure events from the 23 evaluated patients are used for training, and the remaining 142 seizure events are used for testing. This study adopts a patient-specific seizure detection setting. For each patient, seizure events are first assigned to the training and testing sets in a non-overlapping manner at the event level, and this assignment is completed before any 4 s segmentation or overlap-based augmentation. Specifically, most patients use the earliest available seizure event for training, whereas patients with very short or frequent seizures use additional early events to ensure sufficient ictal training data. All subsequent seizure events are reserved for testing. Note that no segment derived from a testing seizure event is used for training or augmentation. To mitigate class imbalance, training-seizure segments are augmented with 80% overlapping sliding windows with a stride of approximately 204 samples, yielding about five-fold augmentation.

The network is trained with cross-entropy loss and L2 regularization:

(24) $$L=-\frac{1}{M}\sum_{i=1}^{M}\sum_{q=1}^{Q}y_{i,q}\log P_{i,q}+\frac{\lambda }{2}\sum_{\theta \notin \Omega_{\omega }}\theta^{2},$$

where *M* is the number of samples, Q=2 is the number of classes, $y_{i,q}$ is the one-hot label, $P_{i,q}$ is the predicted probability, and λ is the regularization coefficient. The set $\Omega_{\omega }$ contains all cosine convolution frequency parameters, which are excluded from weight decay to avoid degeneration of cosine kernels into constant filters. Table 6 summarizes the training hyperparameters. The learning rate follows a step-decay schedule $\eta_{e}=\eta_{0}\cdot \gamma^{\left\lfloor e/20\right\rfloor }$, where $\gamma ={\left(\eta_{\mathrm{end}}/\eta_{0}\right)}^{1/N_{\mathrm{drops}}}$ and $N_{\mathrm{drops}}=\left\lfloor E_{\max}/20\right\rfloor$. With $E_{\max}=500$, the learning rate decays from $2\times 10^{-4}$ to $2\times 10^{-5}$, balancing early convergence and late-stage refinement. Deterministic training is enabled to ensure reproducibility under identical configurations.

For continuous EEG evaluation, the segment-level seizure probabilities are converted into event-level detections through a postprocessing pipeline comprising score smoothing, thresholding, and collar-based merging, following the procedure described in [26,31,34]. The three postprocessing parameters (i.e., smoothing window *h*, decision threshold θ, and collar length $L_{z}$) are independently optimized for each patient to balance sensitivity and specificity. For each patient, an additional 20 min seizure-free EEG segment was extracted as a calibration dataset, which was used to patient-specifically calibrate the postprocessing parameters (the smoothing window *h*, decision threshold θ, and collar length $L_{z}$) for score smoothing, thresholding, and collar refinement in event-level detection. Importantly, this calibration set was not used for network training and did not update any model weights, and it was strictly separated from both the training and testing sets by selecting non-ictal periods that do not overlap with any seizure events or non-seizure segments used for training/testing and by excluding it from any sample generation or upsampling procedure.

### 3.5. Evaluation Metrics

Model performance was evaluated at both the segment and event levels. At the segment level, TP, FP, TN, and FN denote the numbers of true-positive, false-positive, true-negative, and false-negative EEG segments, respectively. Segment-level sensitivity, specificity, and accuracy are defined as

(25) $$\mathrm{Sensitivity}=\frac{\mathrm{TP}}{\mathrm{TP}+\mathrm{FN}}$$

(26) $$\mathrm{Specificity}=\frac{\mathrm{TN}}{\mathrm{TN}+\mathrm{FP}}$$

(27) $$\mathrm{Accuracy}=\frac{\mathrm{TP}+\mathrm{TN}}{\mathrm{TP}+\mathrm{FP}+\mathrm{TN}+\mathrm{FN}}$$

respectively. These metrics quantify the model’s ability to detect seizure segments, reject normal segments, and correctly classify all segments.

At the event level, a seizure event was considered detected if at least one segment within its annotated seizure interval was classified as a seizure after postprocessing. Event sensitivity was calculated as

(28) $$\mathrm{Event}\;\mathrm{Sensitivity}=\frac{N_{d}}{N_{e}}$$

where $N_{e}$ is the total number of seizure events in the test set, excluding training seizures, and $N_{d}$ is the number of detected events. The false detection rate (FDR) was used to measure false alarms per hour:

(29) $$\mathrm{FDR}=\frac{N_{\mathrm{false}}}{T_{\mathrm{test}}}\;\left(\mathrm{events}/h\right)$$

where $N_{\mathrm{false}}$ is the number of detected events that do not overlap with any true seizure and $T_{\mathrm{test}}$ is the test duration in hours. In addition, the area under the receiver operating characteristic curve (AUC) was calculated by sweeping the decision threshold θ. AUC provides a threshold-independent measure of discrimination between seizure and non-seizure segments. In this study, AUC was computed with fixed postprocessing parameters (h=6, $L_{z}=6$), so that the metric reflects the intrinsic separability of model outputs rather than individual postprocessing optimization.

## 4. Results

This section reports the detection performance of the proposed CosFNet (2-layer cosine convolution with 64 channels per layer and kernel length 9, 1-layer FNet with $d_{\mathrm{ff}}=64$, 19,458 parameters, architecture detailed in Section 2) on both the CHB-MIT and SH-SDU datasets. All experiments employ identical training strategies.

### 4.1. CHB-MIT Results

Table 7 presents the per-patient detection results on the CHB-MIT dataset across 23 patients. Across the 23 evaluated patients, CosFNet achieves a mean segment-level sensitivity of 97.60%, specificity of 97.12%, accuracy of 97.12%, event-level sensitivity of 98.59%, FDR of 0.82/h, and AUC of 97.87%. The patient-wise results indicate that high sensitivity is not restricted to a small subset of subjects: 16 patients reach 100% segment-level sensitivity, and four of the remaining seven patients still exceed 97%. Specificity is also stable, with 16 patients exceeding 97% and nine exceeding 99%, suggesting that the compact model suppresses false detections effectively despite its small parameter count.

The event-level results further support the clinical relevance of the detections. Among 142 test seizure events, 140 are detected, and 21 of 23 patients achieve 100% event-level sensitivity. Missed events occur only in Patients 13 and 18. AUC values are above 95% in 20 patients and above 97% in 19 patients, with Patient 9 reaching 100%, showing that the model outputs retain strong threshold-independent discriminability for most subjects.

The lower results observed in Patients 8, 13, and 18 are consistent with the clinical difficulty of these subjects rather than isolated failures of the proposed architecture. Patient 8 is a very young child, and previous work has also reported low specificity for this patient [35]. Patient 13 has been associated with atypical seizure morphology and an onset zone that may deviate from common scalp electrode coverage [35,48]. Patient 18 has the lowest AUC and highest FDR in this study, and neurological review has reported difficulty in determining the seizure onset location [48]. These cases highlight the remaining challenge of seizure detection under atypical or weakly localized scalp EEG manifestations.

### 4.2. SH-SDU Results

Table 8 presents the per-patient results on the SH-SDU dataset (8 adult patients, ages 28–79). This dataset differs from CHB-MIT in patient demographics, acquisition hardware, and montage configuration (detailed in Section 3.2), serving to validate the robustness of the proposed method across different clinical settings. On the SH-SDU dataset, CosFNet achieves a mean sensitivity of 92.87%, specificity of 94.74%, accuracy of 95.21%, event-level sensitivity of 99.41%, FDR of 1.56/h, and AUC of 96.29%. Although this clinical cohort differs from CHB-MIT in age distribution, montage, and acquisition system, the model maintains robust discrimination. Patients 3 and 7 reach 100% sensitivity, Patient 7 also obtains 100% specificity and accuracy, four patients exceed 98% specificity, and six patients exceed 92% accuracy.

The event-level performance is particularly stable on SH-SDU, with 151 of 154 test seizure events detected. Seven of the eight patients achieve 100% event-level sensitivity, and missed detections occur only in Patient 8. Four patients maintain FDR values no higher than 1.0/h, and Patient 7 has no false detections. The relatively high FDRs in Patients 4 and 5 are consistent with prior clinical observations on the same dataset, where significant EMG artifacts were reported for Patient 4 and high EEG variability with low-amplitude epileptiform features was reported for Patient 5 [35]. These factors may explain the increased false alarm rate under real-world clinical recording conditions.

**Figure 2:**
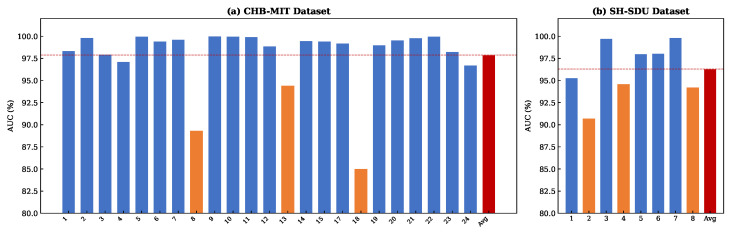
Per-patient AUC distribution. (**a**) CHB-MIT dataset (23 patients). (**b**) SH-SDU dataset (8 patients). The red bar and red dashed line indicate the mean AUC for each dataset.

Figure 2 presents the per-patient AUC distribution across both datasets in bar chart form. In the CHB-MIT dataset, the vast majority of patients achieve AUC above 95%, with only 3 patients falling below this level. In the SH-SDU dataset, all patients achieve AUC above 90%, with 5 out of 8 exceeding 95%.

### 4.3. Training Convergence

**Figure 3:**
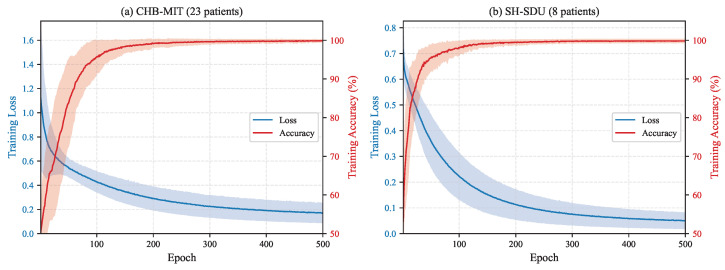
Average cross-entropy training loss and training accuracy curves. (**a**) CHB-MIT dataset (23 patients). (**b**) SH-SDU dataset (8 patients). The shaded area around the curves represents the ±1 standard deviation region.

The average cross-entropy training loss and training accuracy curves for all patients are shown in Figure 3. On the CHB-MIT dataset (23 patients), the average training loss decreased rapidly within the first 100 epochs and stabilized after approximately 300 epochs, while the training accuracy rose to above 99% within 200 epochs and remained stable thereafter. The narrow shaded region indicates low inter-patient variance, suggesting that the model converges consistently across subjects with varying seizure characteristics. On the SH-SDU dataset (8 patients), the training loss exhibited a similar decreasing trend and stabilized after approximately 300 epochs, with the training accuracy reaching comparable levels. The broader standard deviation region reflects larger inter-patient variability in this clinical cohort. These convergence characteristics indicate that CosFNet achieves efficient feature learning through end-to-end optimization with no apparent overfitting, despite containing only 19,458 learnable parameters.

## 5. Discussion

This section discusses the effectiveness and compactness of CosFNet from three perspectives, namely an ablation study, feature visualization, and comparison with existing seizure detection methods.

### 5.1. Ablation Study

To systematically validate the effectiveness of each component and architectural choice in CosFNet, we conduct ablation experiments using the data from Patient 12 in the CHB-MIT dataset. Patient 12 was chosen because its training and test sets contain the largest number of seizure events (23 testing seizures) in the dataset, thereby providing a relatively more sufficient basis for an ablation comparison. All experiments use identical training configurations (500 epochs), and only the target variable is varied in each experiment to ensure a fair comparison. Unless otherwise stated, the proposed configuration is a two-layer cosine convolution with 64 channels (kernel length *K* = 9), one FNet layer (hidden dimension $d_{\mathrm{ff}}$ = 64), and max pooling, totaling 19,458 parameters.

#### 5.1.1. Effectiveness of Key Modules

Table 9 compares the proposed CosFNet with three ablated variants to evaluate the contributions of cosine convolution and FNet. The two models achieve nearly identical AUC values (98.89% and 98.84%), whereas cosine convolution uses only 34.6% of the parameters required by the corresponding standard-convolution model. A similar reduction is observed without FNet, where the CosConv-only model contains 10,882 parameters compared with 47,618 parameters in the StdConv-only model. These results indicate that cosine basis parameterization imposes an effective structural prior on temporal kernels and markedly reduces redundant degrees of freedom without degrading performance on the representative patient. Since EEG is an oscillatory electrophysiological signal, the use of cosine functions also provides a more interpretable kernel form than unconstrained free weights.

The ablation results further show that FNet is particularly important when the convolutional frontend is highly compact. Removing FNet from the cosine convolution model reduces AUC from 98.89% to 93.81%, whereas the corresponding decrease in the standard-convolution model is comparatively small (97.95% vs. 98.84%). This asymmetric effect suggests that cosine convolution and FNet play complementary roles. Cosine convolution compresses local temporal filtering into a small number of learnable parameters, while FNet restores global interaction across temporal positions and hidden features through parameter-free Fourier mixing, with a lightweight feed-forward network supplying nonlinear transformation. In contrast, standard convolution already has substantially more local degrees of freedom and therefore relies less on the FNet encoder.

Beyond the component-level analysis, the following experiments explore how individual architectural variables influence model performance. Five variables are investigated in sequence, namely cosine convolution depth, FNet depth, FNet hidden dimension $d_{\mathrm{ff}}$, classifier head, and channel count. The kernel length is fixed at K=9 throughout. For each experiment, only the named variable is varied while all other settings remain at the proposed configuration.

#### 5.1.2. Effect of Cosine Convolution Depth

To investigate the effect of cosine convolution depth on feature extraction, the number of layers is varied from 1 to 3. As shown in Table 10, AUC increases marginally from one layer to two layers (98.56% vs. 98.89%) and drops notably at three layers (97.28%). The degradation at three layers may reflect increased model complexity, which could raise optimization difficulty or the risk of overfitting under the limited single-patient training data. A two-layer cosine convolution frontend therefore provides the best capacity–trainability balance, consistent with the proposed configuration.

#### 5.1.3. Effect of FNet Depth

To identify how much global mixing depth the compact backbone requires, the number of FNet layers is varied from 0 (no FNet) to 3, with $d_{\mathrm{ff}}$ fixed at 64. As shown in Table 11, removing FNet causes a sharp performance drop (from 98.89% to 93.81%), confirming that global Fourier-domain mixing is essential for the compact cosine convolution frontend. However, increasing FNet depth beyond one layer yields no further improvement (98.73% at 2 layers, 98.82% at 3 layers) despite 44% and 88% more parameters, respectively. This indicates that a single global-mixing pass already supplies sufficient long-range context for the compact backbone, consistent with the proposed configuration.

#### 5.1.4. Effect of FNet Hidden Dimension ($d_{\mathrm{ff}}$)

To assess the impact of FFN capacity, $d_{\mathrm{ff}}$ is varied over {64, 128, 256} with a single FNet layer fixed. As shown in Table 12, increasing the FFN hidden dimension does not improve performance. The smallest setting, $d_{\mathrm{ff}}$ = 64, achieves the highest AUC, whereas larger dimensions introduce additional parameters with slightly lower AUC values. Enlarging $d_{\mathrm{ff}}$ from 64 to 256 raises the parameter count by 127% but reduces AUC from 98.89% to 98.45%. This indicates that a 64-dimensional feed-forward hidden layer is already sufficient to express the post-mixing nonlinearity required by the 64-channel cosine convolution frontend. Additional FFN capacity introduces redundant degrees of freedom without improving detection performance. The smallest $d_{\mathrm{ff}}$ = 64 is therefore retained.

#### 5.1.5. Effect of Classifier Head

To evaluate the effect of the classifier head, four pooling strategies are compared on the same backbone, namely max pooling, mean pooling, attention pooling, and flattening. As shown in Table 13, max pooling preserves the strongest seizure-related response in each feature channel and offers temporal invariance at zero parameter cost, achieving the highest AUC (98.89%). Mean pooling and attention pooling achieve lower AUC values of 97.24% and 97.47%, respectively, indicating that averaging or learned-weight aggregation dilutes localized seizure-relevant peaks. Flattening fails entirely (47.20%): the FNet output of shape [N,256,64] produces a 16,384-dimensional classifier input, inflating the classifier head to 32,768 parameters and causing severe overfitting on the limited training data. Therefore, adopting max pooling in the proposed configuration is reasonable.

#### 5.1.6. Effect of Channel Count

To identify the channel width at which performance saturates, the per-layer cosine convolution channel count is varied over nine scales from 32 to 512. As shown in Table 14, AUC rises sharply from *C* = 48 to *C* = 64 (96.71% vs. 98.89%) and then enters a plateau. For wider models, AUC fluctuates within a narrow range, from 97.68% to 98.23%, across *C* = 96 to *C* = 512, despite a substantial increase in parameter count. These results suggest that 64 channels are sufficient to capture the relevant features. Combined with the lightweight design goal of this work, 64 channels are selected as the channel count for the final model.

### 5.2. Feature Visualization

To intuitively demonstrate the progressive feature extraction process of CosFNet, t-SNE [49] is employed to visualize features at three key stages: (a) raw input EEG, (b) CosConv Layer-2 output, and (c) FNet encoder output. CHB-MIT Patient 12 (275 seizure + 275 normal segments) and SH-SDU Patient 8 (680 seizure + 680 normal segments) serve as representative examples.

**Figure 4:**
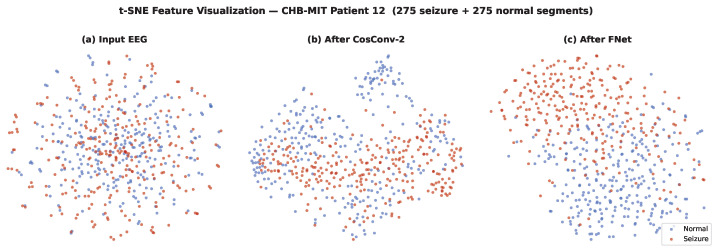
t-SNE feature visualization for CHB-MIT Patient 12. (**a**) Raw input EEG; (**b**) after CosConv Layer-2; (**c**) after FNet encoder. Red indicates seizure segments and blue indicates normal segments.

**Figure 5:**
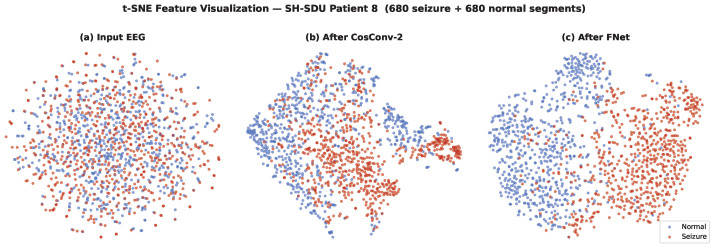
t-SNE feature visualization for SH-SDU Patient 8. (**a**) Raw input EEG; (**b**) after CosConv Layer-2; (**c**) after FNet encoder. Red indicates seizure segments and blue indicates normal segments.

As shown in Figure 4 and Figure 5, seizure and normal samples are highly mixed in the raw EEG embedding, indicating the difficulty of separating the two classes directly from the input space. After the second cosine convolution layer, local clustering begins to emerge, suggesting that the cosine convolutional frontend extracts useful temporal features. The FNet output exhibits clearer separation between seizure and normal samples in both datasets, indicating that global feature mixing further improves the discriminability of the learned representations. This progressive separation is consistent with the ablation results: cosine convolution provides compact local feature extraction, while FNet enhances inter-temporal discrimination through global mixing.

### 5.3. Comparison with Existing Methods

To comprehensively evaluate CosFNet, Table 15 compares its performance with recent seizure detection methods on the CHB-MIT dataset.

As shown in Table 15, CosFNet achieves segment-level sensitivity (97.60%) and specificity (97.12%) that are competitive with state-of-the-art methods. Event-level sensitivity reaches 98.59%, comparable to CosCNN [34] (99.31%), CNN + ViT [28] (98.95%), and MCC-HTSCC [31] (98.61%), indicating near-complete detection of clinical seizure events. FDR of 0.82/h falls within the range of compared methods (0.20–0.98/h).

To further clarify the comparison with the two most closely related cosine-convolution-based methods, we additionally conducted a patient-level statistical comparison on the common 23-patient CHB-MIT cohort after applying the same patient-inclusion criterion as CosFNet (Patient 16 excluded). Paired *t*-tests and Wilcoxon signed-rank tests were conducted between CosFNet and Group CosCNN [35], as well as between CosFNet and MCC-HTSCC [31], followed by Holm correction for multiple comparisons. Compared with Group CosCNN, no statistically significant difference was detected in sensitivity, specificity, event-level sensitivity, FDR, or AUC after correction. Compared with MCC-HTSCC, no statistically significant difference was detected in sensitivity, event-level sensitivity, or FDR. Importantly, CosFNet contains only 19,458 learnable parameters, corresponding to 5.7% of Group CosCNN and 24.3% of MCC-HTSCC.

Among methods reporting parameter counts in Table 15, CosFNet contains only 19,458 parameters, representing merely 7.3% of CosCNN [34] (∼265 K), 5.7% of Group CosCNN [35] (∼340 K), 7.8% of CNN + ViT [28] (∼250 K), and 24.3% of MCC-HTSCC [31] (∼80 K). This parameter reduction is mainly attributed to the compact cosine-kernel representation, the parameter-free FFT mixing operation in FNet, and the use of a 64-channel configuration enabled by the global token-feature mixing capability of the FNet encoder. Therefore, although several reference methods report marginally higher values in specific metrics, CosFNet maintains competitive detection performance with substantially fewer parameters, demonstrating high parameter efficiency and supporting deployment on resource-constrained wearable or embedded EEG-monitoring devices.

## 6. Conclusions

In this work, we proposed CosFNet, a lightweight hybrid architecture for EEG seizure detection. The raw multi-channel EEG recordings were preprocessed and fed into the CosFNet model for end-to-end seizure detection. The designed CosFNet contained a two-layer cosine convolution frontend parameterizing each convolutional kernel with only two learnable parameters (amplitude and frequency) to efficiently capture local spatiotemporal features, and an FNet encoder that replaced self-attention with a parameter-free two-dimensional discrete Fourier transform for global token-feature mixing, with a lightweight feed-forward network providing nonlinear expressiveness. The experimental evaluation on the public CHB-MIT dataset and the clinically collected SH-SDU dataset demonstrated that CosFNet achieved competitive seizure detection performance on both datasets, validating the effectiveness of the proposed method. Comprehensive ablation studies confirmed the complementary synergy between cosine convolution and FNet, and a comparison with existing methods further demonstrated the high parameter efficiency of the proposed architecture, establishing a foundation for deployment on resource-constrained wearable and embedded platforms. In future work, we will further validate the model on larger public datasets and measure inference latency and energy consumption on actual embedded hardware to assess practical deployment feasibility.

## Figures and Tables

**Table 1:** Computational complexity comparison between self-attention and FNet.

Module	Complexity	Learnable Params
Self-Attention	$O(L^{2}D)$	$4D^{2}$ (QKV + projection)
FNet (2D-DFT mixing)	O(LD(logL+logD))	0
FNet (FFN)	$O(L\cdot D\cdot d_{ff})$	$2Dd_{ff}+D+d_{ff}$

Params denotes learnable parameters. QKV denotes query, key, and value projections; 2D-DFT denotes the two-dimensional discrete Fourier transform; and FFN denotes feed-forward network.

**Table 2:** Layer-wise configuration and parameter count of CosFNet.

Layer	Type	Input Shape	Output Shape	Learnable Parameters
1	CosConv Block	B×18×1×1024	B×64×1×512	18×64×2+64×2=2432
2	CosConv Block	B×64×1×512	B×64×1×256	64×64×2+64×2=8320
3	Squeeze + Permute	B×64×1×256	B×256×64	0
4	FNet Encoder (×1)	B×256×64	B×256×64	2×64×64+64+64+4×64=8576
5	Max Pool (temporal)	B×256×64	B×64	0
6	Linear	B×64	B×2	64×2+2=130
	Total			19,458

**Table 3:** CHB-MIT dataset patient information.

Patient	Sex	Age	$N_{u}$	$N_{t}$	Duration (h)	Mean Sz (s)	Train Sz (s)
1	F	11	7	1	40.55	63.15	40
2	M	11	3	1	35.27	57.34	81
3	F	14	7	1	38.00	57.43	52
4	M	22	4	1	156.07	94.50	49
5	F	7	5	1	39.00	111.60	115
6	F	1.5	10	4	66.74	15.30	64
7	F	14.5	3	1	67.05	108.34	86
8	M	3.5	5	1	20.01	183.80	171
9	F	10	4	1	67.87	69.00	64
10	M	3	6	1	50.02	65.50	35
11	F	12	3	1	34.79	268.67	22
12	F	2	27	4	20.69	36.63	129
13	F	3	12	4	33.00	44.59	209
14	F	9	8	1	26.00	21.13	14
15	M	16	20	1	40.01	99.60	125
16 ^†^	F	7	10	8	19.00	8.40	69
17	F	12	3	1	21.01	97.67	90
18	F	18	6	1	35.63	52.84	50
19	F	19	3	1	29.93	78.67	78
20	F	6	8	1	27.60	36.75	29
21	F	13	4	1	32.83	49.75	56
22	F	9	3	1	31.00	68.00	58
23	F	6	7	1	26.56	60.58	113
24	—	—	16	1	21.30	31.94	25
**Sum **	17F/5M	—	**174**	**32**	**960.93**	—	—

$N_{u}$: total expert-annotated seizure events. $N_{t}$: seizure events used for training. Duration: total EEG recording length. Mean Sz: mean seizure duration across all $N_{u}$ events. Train Sz: total duration of training seizures. ^†^ Patient 16 excluded from the Sum row due to extremely short seizure durations (mean 8.40 s). Bold values indicate aggregate values in the Sum row.

**Table 4:** SH-SDU dataset patient information.

Patient	Sex	Age	$N_{u}$	$N_{t}$	Duration (h)	Mean Sz (s)	Train Sz (s)
1	F	28	11	2	23.75	66.82	177
2	M	61	10	2	16.04	220.80	347
3	M	34	10	2	12.00	52.20	94
4	M	72	29	2	15.56	109.38	191
5	M	79	38	3	17.38	68.71	275
6	F	76	3	1	12.00	147.67	137
7	F	38	3	1	6.00	34.67	32
8	M	49	66	3	57.50	115.00	320
**Sum **	3F/5M	—	**170**	**16**	**160.23**	—	—

Column definitions are identical to Table 3. Bold values indicate aggregate values in the Sum row.

**Table 5:** Preprocessing parameter comparison between CHB-MIT and SH-SDU datasets.

Specification	CHB-MIT	SH-SDU
Number of Subjects	24	8
Age Range (years)	1.5–22	28–79
Total Duration (h)	979.93	160.23
Seizure Events	184	170
Montage	18 bipolar	18 unipolar
Original Sampling Rate (Hz)	256	500
Used Sampling Rate (Hz)	256	256 (resampled)
DWT Passband (Hz)	4–32	4–32
Segment Length	4 s (1024 samples)	4 s (1024 samples)
Seizure Oversampling	5× (80% overlap)	5× (80% overlap)
Input Shape	(N,18,1,1024)	(N,18,1,1024)

The CHB-MIT entries in this table report the original public dataset statistics. Patient 16 is excluded from the evaluated cohort in this study, as described in Section 3.1.

**Table 6:** Training hyperparameters.

Parameter	Value
Optimizer	Adam [43]
Initial learning rate $\eta_{0}$	$2\times 10^{-4}$
Final learning rate $\eta_{end}$	$2\times 10^{-5}$
Weight decay λ (excl. ω)	$1\times 10^{-3}$
Batch size	128
Training epochs $E_{max}$	500
LR decay period	20 epochs
Loss function	CrossEntropyLoss

**Table 7:** Per-patient seizure detection results on the CHB-MIT dataset (23 patients).

Patient No.	Segment-Based Evaluation			Event-Based Evaluation				AUC (%)
	Sensitivity (%)	Specificity (%)	Accuracy (%)	$N_{e}$	$N_{d}$	Sensitivity (%)	FDR (/h)	
1	100.00	98.89	98.89	6	6	100.00	0.17	98.35
2	100.00	99.54	99.54	2	2	100.00	0.45	99.82
3	100.00	94.45	94.46	6	6	100.00	1.00	97.94
4	100.00	93.46	93.46	3	3	100.00	1.13	97.11
5	100.00	99.95	99.95	4	4	100.00	0.03	99.97
6	100.00	98.83	98.83	6	6	100.00	1.86	99.43
7	100.00	99.95	99.95	2	2	100.00	0.01	99.62
8	85.42	89.72	89.67	4	4	100.00	0.50	89.32
9	100.00	99.99	99.99	3	3	100.00	0.01	100.00
10	100.00	99.95	99.95	5	5	100.00	0.00	99.97
11	100.00	99.86	99.86	2	2	100.00	0.03	99.92
12	97.07	97.17	97.17	23	23	100.00	1.31	98.87
13	80.00	96.22	96.17	8	7	87.50	2.00	94.40
14	100.00	99.07	99.07	7	7	100.00	0.69	99.48
15	98.35	97.57	97.58	19	19	100.00	0.78	99.43
17	100.00	98.88	98.88	2	2	100.00	0.19	99.20
18	86.11	83.93	83.93	5	4	80.00	4.10	84.99
19	100.00	99.20	99.20	2	2	100.00	0.10	98.98
20	98.63	97.40	97.40	7	7	100.00	1.45	99.54
21	100.00	98.66	98.67	3	3	100.00	0.98	99.80
22	100.00	99.99	99.99	2	2	100.00	0.03	99.98
23	100.00	96.76	96.77	6	6	100.00	0.90	98.25
24	99.26	94.32	94.36	15	15	100.00	1.17	96.70
**Summary **	**97.60**	**97.12**	**97.12**	**142**	**140**	**98.59**	**0.82**	**97.87**

$N_{e}$: number of seizure events in the test set. $N_{d}$: number of events detected by the model. Patient 16 excluded (see Table 3). Bold values indicate the cohort-level summary row. Segment-level metrics, FDR, and AUC are reported as patient-wise means; $N_{e}$ and $N_{d}$ are totals, and event-level sensitivity is calculated as $N_{d}/N_{e}$.

**Table 8:** Per-patient seizure detection results on the SH-SDU dataset (8 patients).

Patient No.	Segment-Based Evaluation			Event-Based Evaluation				AUC (%)
	Sensitivity (%)	Specificity (%)	Accuracy (%)	$N_{e}$	$N_{d}$	Sensitivity (%)	FDR (/h)	
1	92.41	90.68	92.52	9	9	100.00	3.21	95.29
2	89.85	98.18	97.09	8	8	100.00	0.25	90.68
3	100.00	98.95	99.30	8	8	100.00	1.00	99.72
4	86.57	91.51	92.10	27	27	100.00	1.87	94.58
5	94.75	93.50	94.79	35	35	100.00	3.24	98.00
6	92.31	98.15	97.48	2	2	100.00	0.42	98.03
7	100.00	100.00	100.00	2	2	100.00	0.00	99.81
8	87.06	86.96	88.36	63	60	95.24	2.53	94.20
**Summary**	**92.87**	**94.74**	**95.21**	**154**	**151**	**99.41**	**1.56**	**96.29**

$N_{e}$: number of seizure events in the test set. $N_{d}$: number of events detected by the model. Bold values indicate the cohort-level summary row. Segment-level metrics, FDR, and AUC are reported as patient-wise means; $N_{e}$ and $N_{d}$ are totals, and event-level sensitivity is calculated as $N_{d}/N_{e}$.

**Table 9:** Component ablation results.

Model	Convolution	FNet	Params	AUC (%)
CosFNet (proposed)	Two-layer CosConv	1 layer ($d_{\mathrm{ff}}$ = 64)	19,458	98.89
CosConv only	Two-layer CosConv	None	10,882	93.81
StdConv + FNet	Two-layer StdConv	1 layer ($d_{\mathrm{ff}}$ = 64)	56,194	98.84
StdConv only	Two-layer StdConv	None	47,618	97.95

**Table 10:** Effect of cosine convolution depth.

Depth (Layers)	Params	AUC (%)
1 layer	11,138	98.56
**2 layers **	**19,458**	**98.89**
3 layers	27,778	97.28

Bold values indicate the configuration adopted in the final CosFNet model.

**Table 11:** Effect of FNet depth.

FNet Layers	Params	AUC (%)
0 (no FNet)	10,882	93.81
**1 **	**19,458**	**98.89**
2	28,034	98.73
3	36,610	98.82

Bold values indicate the configuration adopted in the final CosFNet model.

**Table 12:** Effect of FNet hidden dimension $d_{\mathrm{ff}}$.

$d_{\mathrm{ff}}$	Params	AUC (%)
**64 **	**19,458**	**98.89**
128	27,714	98.58
256	44,226	98.45

Bold values indicate the configuration adopted in the final CosFNet model.

**Table 13:** Effect of classifier head.

Head	Params	AUC (%)
**Max pooling **	**19,458**	**98.89**
Mean pooling	19,458	97.24
Attention pooling	19,523	97.47
Flatten	52,098	47.20

Bold values indicate the configuration adopted in the final CosFNet model.

**Table 14:** Effect of channel count.

Channels	Params	AUC (%)
32	7,714	96.18
48	13,074	96.71
**64 **	**19,458**	**98.89**
96	35,298	97.81
128	55,234	98.14
192	107,394	98.02
256	175,938	98.23
384	362,178	97.69
512	613,954	97.68

Bold values indicate the configuration adopted in the final CosFNet model.

**Table 15:** Performance comparison with recent methods on the CHB-MIT dataset.

No.	Author (Year)	Method	Params	Train Sz	Sens (%)	Spc (%)	EvtSens (%)	FDR (/h)
1	Li et al. (2020) [25]	FC-NLSTM	—	63	95.42	95.29	94.07	0.66
2	Li et al. (2021) [10]	EMD + CSP + SVM	—	54	97.34	97.50	98.47	0.63
3	Wang et al. (2021) [16]	1D-CNN	—	121	88.14	99.62	99.31	0.20
4	Zhang et al. (2022) [40]	BiGRU	—	73	93.89	98.49	95.49	0.31
5	Zhong et al. (2024) [50]	ST + Trans.	—	64	96.11	96.38	96.57	0.38
6	Dong et al. (2024) [26]	TCN + BiLSTM	—	56	94.31	97.13	96.48	0.38
7	Li et al. (2024) [51]	Sup. Contrastive	—	80	98.97	97.36	99.71	0.35
8	Liu et al. (2024) [34]	CosCNN	∼265 K	40	98.12	98.19	99.31	0.69
9	Liu et al. (2025) [35]	Group CosCNN	∼340 K	24	97.70	97.54	98.61	0.98
10	Wang et al. (2025) [28]	CNN + ViT	∼250 K	40	98.09	98.21	98.95	0.31
11	Chen et al. (2026) [31]	MCC-HTSCC	∼80 K	—	97.98	98.53	98.61	0.95
12	**This work **	**CosFNet**	∼**19.5K**	**32**	**97.60**	**97.12**	**98.59**	**0.82**

Params denotes the approximate number of learnable parameters when reported or clearly derivable from the original publication. Train Sz denotes the number of seizure events used for training. Values are collected from the corresponding original publications under their reported experimental settings. Bold type identifies the proposed CosFNet method (this work).

## Data Availability

The CHB-MIT dataset is publicly available. The SH-SDU dataset used in this study can be made available on request.
